# Concurrent Use of Oral Anticoagulants and Sulfonylureas in Individuals With Type 2 Diabetes and Risk of Hypoglycemia: A UK Population-Based Cohort Study

**DOI:** 10.3389/fmed.2022.893080

**Published:** 2022-08-23

**Authors:** Hassan Alwafi, Ian C. K. Wong, Abdallah Y. Naser, Amitava Banerjee, Pajaree Mongkhon, Cate Whittlesea, Alaa Alsharif, Li Wei

**Affiliations:** ^1^Research Department of Practice and Policy, School of Pharmacy, University College London, London, United Kingdom; ^2^Faculty of Medicine, Umm Al Qura University, Mecca, Saudi Arabia; ^3^Centre for Safe Medication Practice and Research, Department of Pharmacology and Pharmacy, Li Ka Shing Faculty of Medicine, The University of Hong Kong, Pokfulam, Hong Kong SAR, China; ^4^Department of Pharmacy, The University of Hong Kong - Shenzhen Hospital, Shenzhen, China; ^5^Laboratory of Data Discovery for Health, Hong Kong Science Park, Pak Shek Kok, Hong Kong SAR, China; ^6^Centre for Medicines Optimisation Research and Education, University College London Hospitals National Health Service (NHS) Foundation Trust, London, United Kingdom; ^7^Department of Applied Pharmaceutical Sciences and Clinical Pharmacy, Faculty of Pharmacy, Isra University, Amman, Jordan; ^8^Institute of Health Informatics, University College London, London, United Kingdom; ^9^Department of Cardiology, University College London Hospitals NHS Trust, London, United Kingdom; ^10^Department of Cardiology, Barts Health NHS Trust, London, United Kingdom; ^11^Department of Pharmacy Practice, School of Pharmaceutical Sciences, University of Phayao, Phayao, Thailand; ^12^Pharmacoepidemiology and Statistics Research Center (PESRC), Faculty of Pharmacy, Chiang Mai University, Chiang Mai, Thailand

**Keywords:** oral anticoagulants, hypoglycemia, sulfonylureas, diabetes mellitus, United Kingdom, drug-drug interactions

## Abstract

**Objective:**

To investigate the association of concurrent use of oral anticoagulants (OACs) and sulfonylureas and the risk of hypoglycemia in individuals with type 2 diabetes mellitus (T2DM).

**Research Design and Methods:**

A retrospective cohort study was conducted between 2001 and 2017 using electronic primary healthcare data from the IQVIA Medical Research Data (IMRD) that incorporates data supplied by The Health Improvement Network (THIN), a propriety database of Cegedim SA. Individuals with T2DM who received OAC prescription and sulfonylureas were included. We compared the risk of hypoglycemia with sulfonylureas and OACs using propensity score matching and Cox regression.

**Results:**

109,040 individuals using warfarin and sulfonylureas and 77,296 using direct oral anticoagulants (DOACs) and sulfonylureas were identified and included. There were 285 hypoglycemia events in the warfarin with sulfonylureas group (incidence rate = 17.8 per 1,000 person-years), while in the sulfonylureas only, 304 hypoglycemia events were observed (incidence rate = 14.4 per 1,000 person-years). There were 14 hypoglycemic events in the DOACs with sulfonylureas group (incidence rates = 14.8 per 1,000 person-years), while in the sulfonylureas alone group, 60 hypoglycemia events were observed (incidence rate =23.7 per 1,000 person-years). Concurrent use of warfarin and sulfonylureas was associated with increased risk of hypoglycemia compared with sulfonylureas alone (HR 1.38; 95% CI 1.10–1.75). However, we found no evidence of an association between concurrent use of DOACs and sulfonylureas and risk of hypoglycemia (HR 0.54; 95% CI, 0.27–1.10) when compared with sulfonylureas only.

**Conclusions:**

We provide real-world evidence of possible drug-drug interactions between warfarin and sulfonylureas. The decision to prescribe warfarin with coexistent sulfonylureas to individuals with T2DM should be carefully evaluated in the context of other risk factors of hypoglycemia, and availability of alternative medications.

## Background

Individuals with type 2 diabetes mellitus (T2DM) are often suffering from cardiovascular complications (1). Oral anticoagulant medications (OACs) including warfarin and direct oral anticoagulants (DOACs) are widely prescribed for the prevention and treatment of stroke, atrial fibrillation (AF) and venous thromboembolism (VTE) (2). However, their use may be associated with a high probability of drug-drug interactions, and serious adverse events (3).

Hypoglycemia is a common complication of antidiabetic medications such as sulfonylureas (4). Sulfonylureas act by lowering the blood glucose level by increasing insulin secretion in the pancreas and by blocking the ATP-sensitive potassium channels (5). Hypoglycemia can be potentiated by several risk factors including drug-drug interaction *via* inhibition of hepatic cytochrome P450 (CYP) enzymes which are responsible for the metabolism of sulfonylureas (6). Drugs that inhibit CYP450 may increase the concentration of sulfonylureas in the circulation and hence increase the risk of hypoglycemia (7). Previous pre-clinical studies have reported a possible drug-drug interaction between warfarin and sulfonylureas through the displaced plasma protein binding and hepatic metabolism CY450 (8–10). Two previous studies reported a significant association between warfarin and sulfonylurea (11, 12), However, both studies used a self-controlled series design, where a major limitation of this design is that patients health status may be different during the exposure period, and patients are sicker in periods of the exposure (13). Besides, Romley et al. only included patients aged >65 years old in their study, and therefore, their results may not be generalized to the entire populations (11), and they did not investigate the association of the concurrent use of sulfonylureas and DOACs.

Given the fact that diabetes and AF are highly prevalent, and that antidiabetic and anticoagulant medications are largely prescribed concomitantly and the possibility of developing drug-drug interactions (3, 14, 15), and the lack of published evidence, we aimed to investigate the association between concurrent OAC and sulfonylurea use and risk of hypoglycemia.

## Research Design and Methods

### Study Design

This was a population-based cohort study in individuals with T2DM from 2001 to 2017.

### Data Source

We used the electronic primary healthcare data from the IQVIA Medical Research Data (IMRD) that incorporates data supplied by The Health Improvement Network (THIN), a propriety database of Cegedim SA for this study. THIN is a UK primary care database containing anonymized administrative, clinical, and prescribing data from over 587 practices with more than 13 million individuals (16, 17). THIN is one of the largest sources for primary care data in the UK and has been validated for epidemiological research purposes (14, 15, 17). It holds data on personal information, health related behaviors, and diagnoses information which is recorded and identified using Read codes. Read codes are coded clinical terminologies that have been used to define the care, diagnosis and the management of diseases. It is used by the NHS to in the UK manage primary care data in electronic health records (18). THIN contains records of prescriptions issued only by GPs and recorded in the individual's records (16, 17). Data from practices that met the acceptable mortality reporting (AMR) measures of quality assurance for THIN data were used in this study (19).

This study was reviewed, and scientific approval was obtained by THIN SRC in 2018 (18THIN046). The research was reported in accordance with strengthening the reporting of observational studies in epidemiology (STROBE) Statement.

### Study Cohort

The study population included people aged at least 18 years old, with a recorded diagnosis of T2DM. Patients with T2DM were identified using the Read codes, which were intensively used in the previous diabetes related studies. To ensure accurate measurement of medical history, we included individuals only if they had an observation period of at least 12 months before the first T2DM diagnosis and were registered with the general practice during the study period. Individuals were followed up until the end of September 2017 and were censored if they experienced the outcome of interest, died, left their general practice during the study period, or stopped medications during the overlapping period of both medications, whichever came first.

**Figure 1:**
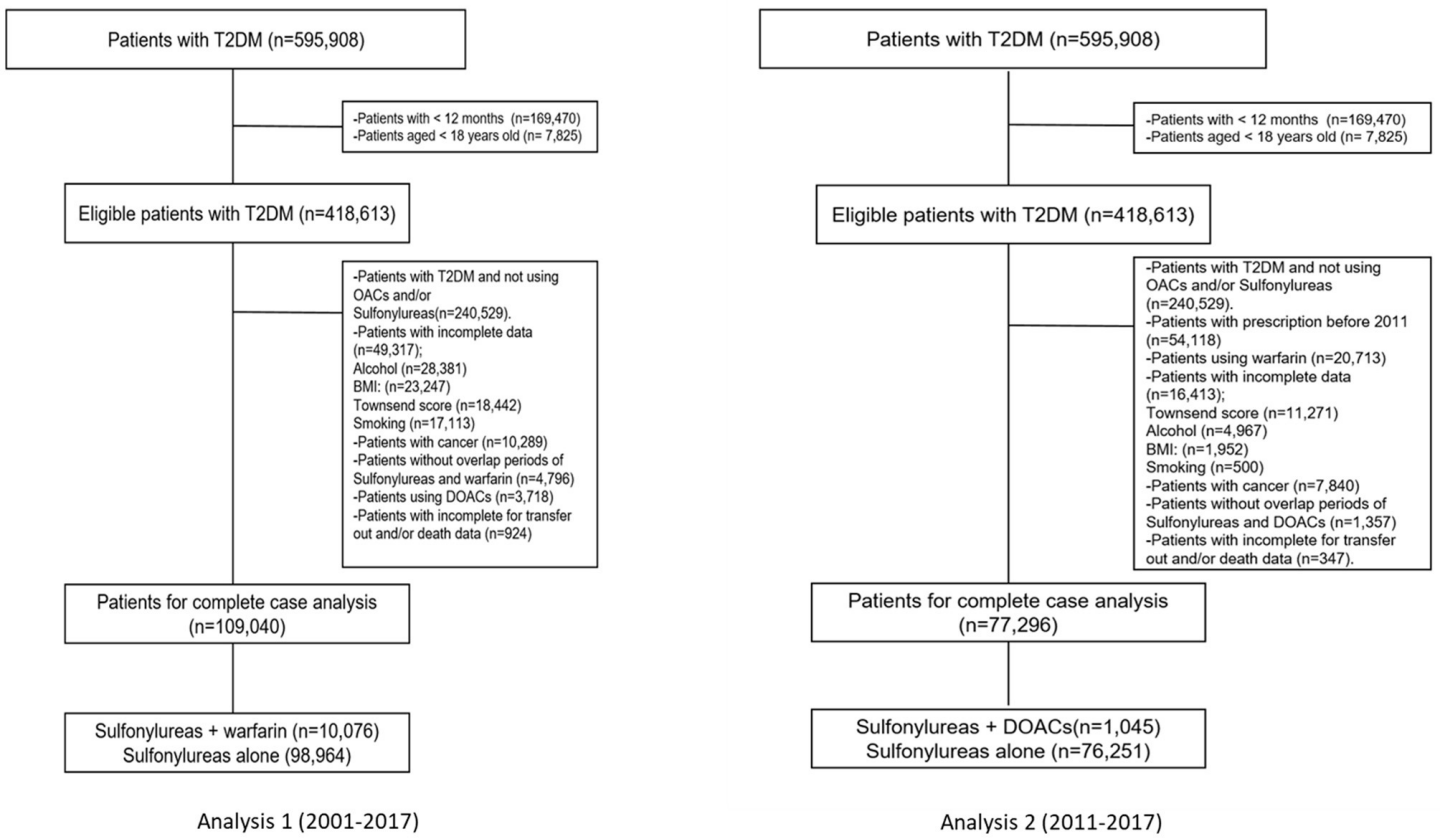
Flow chart of the study cohorts.

The study covered a period of 17 calendar years (2001–2017), and two separate but similar analyses were carried out. The first and second analyses comprised individuals with T2DM who received at least one prescription for one of the OACs of interest [including warfarin (Analysis 1) or DOACs (Analysis 2)] and sulfonylureas and were identified using drug codes recorded in THIN. The index date (start date) was defined as the date on which users were first co-exposed (concurrently) to both medications (OACs and sulfonylureas), regardless of which medication was first. Individuals with records of prescriptions of both medications during the follow up were included in the study. The index date was defined as: (a) if sulfonylurea prescription was between the first and last warfarin prescriptions then the date of sulfonylurea was accounted as the index date if warfarin prescription was between the first and last sulfonylurea prescriptions then the date of warfarin was accounted as the index date. Individuals who did not meet these criteria were categorized as ‘no overlap' and excluded. We also excluded individuals if they had <12 months observation period, were <18 years old, were diagnosed with malignancy or metastatic tumors (as they are at higher risk of glucose level fluctuations due to anticancer therapy) and individuals with incomplete data for transfer out or death data. Details of the exclusion criteria are provided in Figure 1.

### Exposure Definition

The exposure of interest was person-time concomitantly exposed to OACs and sulfonylureas after the diagnosis of T2DM. OACs included warfarin and DOACs (dabigatran, apixaban, rivaroxaban and edoxaban). Individuals with records of acenocoumarol or phenindione only were not included, because of the very low number of individuals and because it is very unlikely to be prescribed in the UK. Sulfonylureas were of a second generation and included: gliclazide, glipizide, glyburide and glimepiride. For Analysis 1, we compared individuals using warfarin and sulfonylureas concurrently, vs. individuals using sulfonylureas only, and the index date for this group was the first prescription of sulfonylureas. For Analysis 2, we compared individuals using DOACs and sulfonylureas concurrently, vs. individuals using sulfonylureas only, and the index date for this group was the first prescription of sulfonylureas. only individuals who were new users from 2011 onwards were included in Analysis 2, as DOACs were first approved on the market from 2011. Further exploratory subgroup analyses were also conducted to investigate the risk of hypoglycemia when warfarin was used with different types of sulfonylureas (gliclazide, glipizide, glyburide and glimepiride).

### Study Outcomes

The study outcome was incident hypoglycemia during the follow up time defined as the first record of hypoglycemia after the index date.

### Study Covariates

Demographics, comorbidities, and medications associated with developing hypoglycemia were included as covariates, based on previous studies (11, 12, 20) and clinician recommendation. These covariates include age, gender, smoking status (never-smoked, ex-smoker and current smoker), body mass index (BMI), alcohol consumption (never-drink, ex-drinker and current drinker), Townsend deprivation score, CVDs disease, history of hyperglycemia, hyperlipidemia, hypertension (HTN), AF, stroke/ transient ischemic attack (TIA), deep vein thrombosis (DVT), peripheral vascular disease (PVD), chronic kidney disease (CKD), liver disease, anxiety, depression, chronic obstructive pulmonary disease (COPD), use of multiple antidiabetics (intensification), beta-blockers (BBs), angiotensin converting enzyme inhibitor (ACEIs), angiotensin II receptor blocker inhibitor (ARBs), calcium channels blockers (CCBs), statins, corticosteroids, antiplatelets. Information for comorbidities was evaluated at any time on/or before the index date and was identified using the Read codes and alcohol consumption, smoking status were the latest records prior to the study entry date. Medications were identified using the drug codes within 180-days on/or before the start date. Townsend deprivation is a measure of material deprivation which is calculated using census data and linked to area of residence in the UK. It includes information for unemployment, overcrowding, car ownership, and home ownership for small geographies, which is calculated to generate an overall score. This is recorded in THIN database as quintiles, with quintile 1 as the lowest (least deprived) and 5, the highest (21). We did not account for international normalized ratio (INR) results because the data was not complete.

### Propensity Score Matching

To minimize potential bias due to non-randomized, a propensity score matching cohort (within ±0.05, comprising 1:1 controls were created (22). To address confounding by indication, when individuals with T2DM with more severe illness are likely to be treated with insulin, sulfonylurea or multiple antidiabetic medications or individuals with T2DM using OACs might be prescribed OACs for different indications, we included the following variables (AF, DVT and stroke/TIA) in the PS model (23). We used a logistic regression model to estimate the propensity score for each individual and variables listed in the previous section were included. Absolute Standardized Differences (ASD) for all baseline variables were calculated to assess the differences in individuals' characteristics and covariates balance between treatment groups before and after the propensity score matching. We used a cut-off point of 0.1 ASD (24–28).

### Statistical Analysis

Individual's characteristics were presented as number (percentage) for categorical variables and as mean (±SD) for continuous variables. Crude incidence rates were calculated by dividing the number of hypoglycemia events by person-time at risk and were expressed as rates per 1,000 person-years. Person-year was calculated as the time from the index date until the end of follow up. The Cox proportional hazard model before and after propensity score was used to estimate the time to an event, and to investigate the association between the use of OACs with co-existent sulfonylurea and the risk of hypoglycemia; results were presented as HR with 95% confidence interval (CI). We performed Kaplan–Meier survival curves plots on the matched datasets to compare outcomes between the cohorts over time. The hazard assumption was examined by both visual graphs and by applying tests using ph statement (proc PHREG) in SAS statistical software. All analysis was performed using SAS version 9.4 (SAS Institute).

### Sensitivity Analysis

To confirm the robustness of our results we conducted five sensitivity analyses. First, we re-analyzed the data taking into account subsequent changes in the exposure status. Individuals were censored if they stopped the treatment. The gap between expected prescription end date and the start date of any subsequent prescription were no more than 90 days.

Second, we repeated the analysis using the IPTW method to address the risk difference between groups at baseline (29). The stabilized IPTW was calculated by multiplying the IPTW by the marginal probability of receiving the actual treatment (29). Stabilization was used to reduce the variability of the IPTW weights (30). The predictor variables inserted in the IPTW models included the same covariates as in the PS matching. PS trimming for the IPTW was also conducted. We re-analyzed the IPTW based on (1st percentile of the PS distribution in exposure and the 99th percentile of the PS in non-exposed treated) (31). Third, the missing data on smoking status, BMI, alcohol consumption and Townsend scores were imputed by the multiple imputation (MI) method (32, 33). All PS covariates, the outcome and survival time were included in the MI model, and 25 imputed datasets were generated, analyzed separately and then combined using Rubin's rule.

### Patient and Public Involvement

It was not appropriate or possible to involve individuals or the public in the design, or conduct, or reporting, or dissemination plans of this research.

### Data and Resource Availability

Data access is through permission from THIN only.

## Results

### Individuals' Characteristics

A total of 418,613 individuals with T2DM were identified, of whom only 178,084 received a prescription for sulfonylureas and OACs at some point during the study period between 2001 and 2017. We excluded 49,317 (22%) individuals in the warfarin group (Analysis 1) and 16,413 (17.5%) individuals in the DOACs group (Analysis 2) for missing data in the main analysis. Finally, after we applied exclusion criteria, 109,040 using warfarin and sulfonylureas (Analysis 1) and 77,296 using DOACs and sulfonylureas (Analysis 2) were included for the analyses. Details of the identification of the study cohort, including the study cohort of individuals included in Analyses 1 and 2 are presented in Figure 1.

At baseline (Table 1), before propensity score matching, compared to individuals who received sulfonylureas only, individuals who received warfarin with sulfonylureas were older (mean age: 73.3 vs. 61.0), had a higher cardiovascular profile: CVDs (14.7 vs. 4.5%), HTN (68 vs. 50%), stroke/TIA (19 vs. 5.7%), AF (61 vs. 1.8%), and DVT (21 vs. 2%). At least 50% of the study population had a BMI ≥30, with nearly similar BMI ratios in individuals who received warfarin with sulfonylureas compared to individuals who received sulfonylureas only. However, gender and the mental health profile including depression and anxiety showed little difference between the two groups (61% females vs. 57%females, 20 vs. 23% and 13 vs. 15%, respectively). After matching, all baseline individuals' characteristics had standardized differences <0.1 (Table 1). Details of the study characteristics, including individuals' characteristics of Analysis 2 (DOACs with sulfonylureas vs. sulfonylureas), are presented in Supplementary Table S1.

**Table 1:** Individuals' characteristics among the cohort of first analysis (sulfonylureas and warfarin vs. sulfonylureas only).

**Variable**	**Before propensity score matching No. (%) of participant**			**After propensity score matching No. (%) of participant**		
	**Sulfonylureas**	**Sulfonylureas**	**Crude**	**Sulfonylureas**	**Sulfonylureas**	**Matched**
	**+ warfarin**	**(*n* = 98,964)**	**ASD**	**+ warfarin**	**(*n* = 5,379)**	**ASD**
	**(*n* = 10,076)**			**(*n* = 5,379)**		
Demographics						
Age mean (SD)	73.3 (10.0)	61.0 (12.8)	0.918	69.9 (10.8)	70.0 (11.8)	0.054
Male, *n* (%)	6,918 (61.1)	63,965 (57.0)	0.083	3,114 (57.89)	3,041 (56.53)	-0.027
BMI			0.037			0.038
BMI <25	1,715 (15.5)	18,491 (16.5)	–	877 (16.3)	923 (17.1)	–
BMI 25–30	3,942 (34.8)	38,351 (34.2)	–	1,837 (34.1)	1,894 (35.2)	–
BMI ≥ 30	5,665 (50.0)	55,341 (49.3)	–	2,665 (49.5)	2,562 (47.6)	–
Smoking			0.228			-0.011
Non-smokers	10,068 (88.9)	90,649 (80.8)	–	4,673 (86.8)	4,652 (86.4)	–
Smokers	1,254 (11.0)	21,534 (19.2)	–	706 (13.3)	727 (13.5)	–
Alcohol			0.035			0.006
Non-drinker	3,604 (31.8)	33,899 (30.2)	–	1,740 (32.3)	1,757 (32.6)	–
Drinker	7,718 (68.2)	78,284 (69.8)	–	3,639 (67.7)	3,622 (67.3)	–
Townsend			0.053			0.036
1 (least deprived)	2,026 (20.1)	19,174 (19.4)	–	1,075 (19.9)	1,021 (19.0)	–
2	2,140 (21.2)	19,555 (19.7)	–	1,091 (20.3)	1,057 (19.6)	–
3	2,168 (21.6)	21,773 (22.0)	–	1,149 (21.4)	1,203 (22.4)	–
4	2,167 (21.5)	21,523 (21.7)	–	1,199 (22.3)	1,222 (22.7)	–
5 (most deprived)	1,575 (15.6)	16,939 (17.2)	–	865 (16.0)	876 (16.3)	–
Comorbid conditions, *n* (%)						
CVDs	1,670 (14.7)	5,039 (4.5)	0.353	684 (12.7)	656 (12.2)	-0.016
Hypertension	7,693 (68.0)	56,550 (50.4)	0.363	3,399 (63.2)	3,370 (62.6)	0.011
Stroke/TIA	2,154 (19.0)	6,429 (5.7)	0.412	838 (15.6)	866 (16.1)	-0.014
Bleeding	2,112 (18.6)	15,302 (13.6)	0.137	1,009 (18.7)	970 (18.0)	-0.019
Hyperlipidemia	2,652 (23.4)	19,747 (17.6)	0.144	1,187 (22.0)	1,194 (22.2)	−0.003
AF	6,952 (61.4)	1,973 (1.8)	1.673	1,862 (34.6)	17,50 (32.5)	0.044
DVT	2,403 (21.2)	2,300 (2.0)	0.627	1,453 (27.0)	1,571 (29.2)	-0.049
Chronic kidney disease	3,016 (26.6)	10,991 (9.8)	0.447	1,099 (20.4)	1,105 (20.5)	−0.003
COPD	1,208 (10.7)	5,081 (4.5)	0.233	481 (9.0)	518 (9.6)	-0.024
Hyperglycemia	422 (3.7)	3,451 (3.0)	0.036	212 (3.9)	216 (4.0)	-0.004
Liver diseases	59 (0.5)	678 (0.6)	0.011	36 (0.7)	49 (0.9)	-0.027
Depression	2,323 (20.5)	25,934 (23.1)	0.063	1,202 (22.3)	1,254 (23.3)	-0.023
Anxiety	1,542 (13.6)	17,554 (15.6)	0.057	811 (15.0)	843 (15.7)	-0.017
Baseline medication use, *n* (%)						
Aspirin use	4,264 (37.6)	34,978 (31.8)	0.137	2,223 (41.3)	2,480 (46.1)	-0.096
Antiplatelet drugs use	716 (6.3)	3,636 (3.2)	0.145	368 (6.8)	394 (7.3)	-0.019
Beta blockers use	5,264 (46.5)	23,663 (21.0)	0.558	1,943 (36.1)	36.12 (38.8)	−0.055
ACEs /ARBs use	8,061 (71.2)	50,245 (44.8)	0.555	3,337 (62.0)	3,328 (61.9)	0.003
Corticosteroids use	1,276 (11.2)	6,854 (6.1)	0.184	578 (10.7)	616 (11.4)	-0.022
Multiple antidiabetic medications use (intensification)	7,705 (68.0)	76,835 (68.5)	0.009	3,559 (66.1)	3,425 (63.7)	0.052

### Hypoglycemia

#### Warfarin With Sulfonylureas vs. Sulfonylureas

There were 578 hypoglycemia events in the warfarin and sulfonylureas group (crude incidence rates = 16.7 per 1,000 person-years), while 7,307 hypoglycemia events were recorded in the sulfonylureas only group with a total follow up of 526,422.49 person-years, (crude incidence rates = 13.8 per 1,000 person-years). The risk of developing hypoglycemia was higher for individuals receiving warfarin with sulfonylureas compared to individuals receiving sulfonylureas alone (HR 1.20; 95% CI, 1.10 – 1.30), *P* < 0.0001) (Table 2).

**Table 2:** Number of events, incidence rates and crude HR, for risk of hypoglycemia.

**Exposure group**	**No. of event**	**Person-years at risk, year**	**IR, per 1,000 person-years (95% CI)**	**Crude HR (95% CI)**
Sulfonylurea + warfarin (*n* = 10,076)	578	34422.40	16.7	1.20 (1.10–1.30)
Sulfonylurea only (*n* = 98,964)	7,307	526422.49	13.8	
Sulfonylurea + DOAC* (*n* = 1,045)	14	956.9	15.0	0.66 (0.39–1.11)
Sulfonylurea only* (*n* = 76,251)	4,514	246345.6	18.0	

* *This analysis included individuals only from 2011 onward*.

#### DOACs With Sulfonylureas vs. Sulfonylureas

Individuals using DOACs with sulfonylureas concomitantly contributed to a total of 957 person-years, during which 14 hypoglycemic events were recorded (crude incidence rates = 15.0 per 1,000 person-years), while in the (sulfonylureas only group, a total of 246,345.65 person-years, with 4,514 hypoglycemia events were recorded (crude incidence rates = 18.0 per 1,000 person-years). The risk of developing hypoglycemia was lower for individuals receiving DOACs with sulfonylureas compared to individuals receiving sulfonylureas alone. However, this was not statistically significant (HR 0.66; 95% CI, 0.39–1.11, *p* = 0.118) (Table 2). In the subgroup analysis, patients who received gliclazide with warfarin had a higher risk of hypoglycemia compared to patients who received gliclazide alone (HR 1.23; 95% CI, 1.12–1.34, *P* = <0.0001). In addition, patients who received glyburide with warfarin had a higher risk of hypoglycemia compared to patients who received glyburide alone (HR 1.82; 95% CI, 1.30–2.52, *P* = <0.0001). However, patients who received either glipizide or glimepiride with warfarin did not have a higher risk of hypoglycemia compared to patients who received either glipizide or glimepiride alone. For details, please see Supplementary Tables S5, S6.

### Propensity Score Matched Analysis

#### Warfarin With Sulfonylureas vs. Sulfonylureas

**Figure 2:**
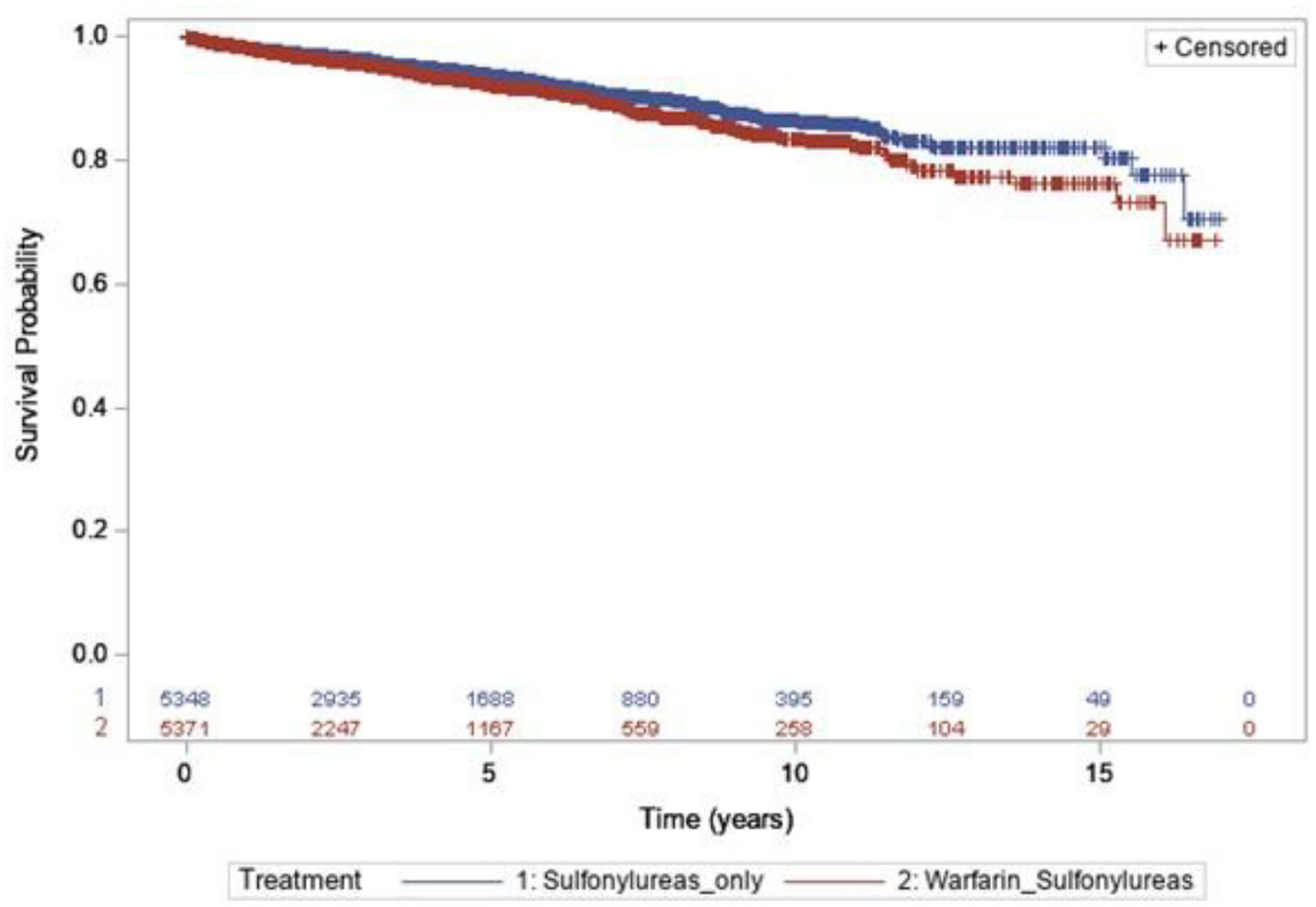
Kaplan–Meier curves for the incidence of hypoglycemia during the follow-up period [sulfonylureas and warfarin (blue line) vs. sulfonylureas only (red line)].

After matching, 5,379 individuals were included in each group. There were a total of 15,959.04 of concomitant exposure and, 285 hypoglycemia events in the warfarin with sulfonylureas group (incidence rates = 17.8 per 1,000 person-years), while users of sulfonylureas only contributed to 21,028.52 person-years, during which 304 hypoglycemia events were observed (incidence rates = 14.4 per 1,000 person-years). The risk of hypoglycemia was 38% higher in individuals with concomitant use of warfarin with sulfonylureas, compared to sulfonylureas alone users (HR 1.38; 95% CI, 1.10–1.76, *P* = 0.010) (Table 3). Kaplan–Meier curves for the incidence of hypoglycemia of Analysis 1 are shown in Figure 2.

**Table 3:** Number of events, incidence rates and HR, for risk of hypoglycemia for the matched cohort.

**Exposure group**	**No. of event**	**Person-years at risk, year**	**IR, per 1,000 person-years (95% CI)**	**Matched HR (95% CI), *p*-value**
Sulfonylurea + warfarin (*n* = 5,379)	285	15959.04	17.8	1.38 (1.10–1.75)
Sulfonylurea only (*n* = 5,379)	304	21028.52	14.4	1.00
Sulfonylurea + DOAC* (*n* = 1,027)	14	942.2	14.8	0.54 (0.27–1.10)
Sulfonylurea only* (*n*=1,027)	60	2532.0	23.7	1.00

* *This analysis included individuals only from 2011 onward*.

#### DOACs With Sulfonylureas vs. Sulfonylureas

**Figure 3:**
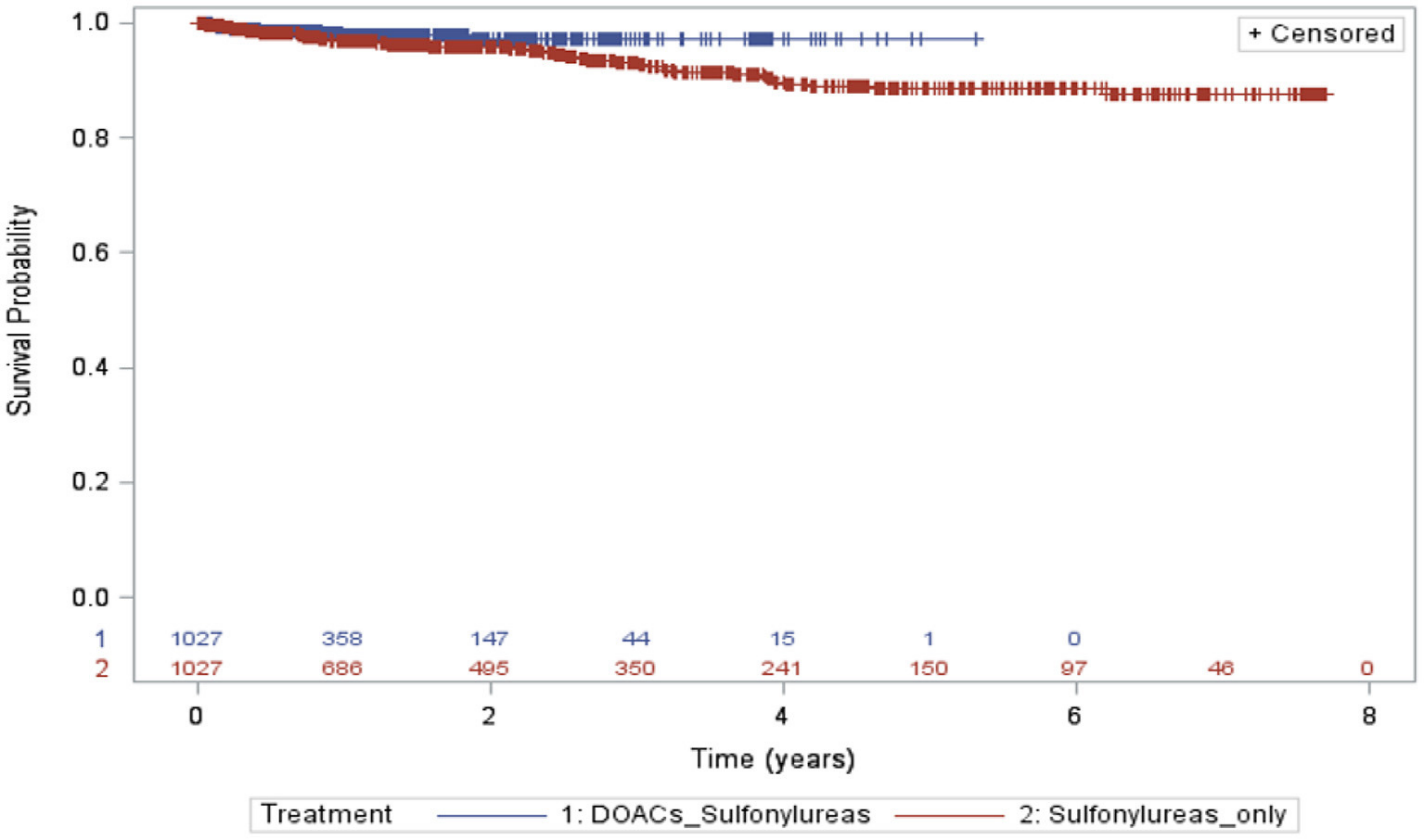
Kaplan–Meier curves for the incidence of hypoglycemia during the follow-up period (sulfonylureas and DOACs vs. sulfonylureas only).

A total of 1,027 in each group were included in the analysis, with a total of 942, and 2,532 person-years were recorded, 14 and 60 hypoglycemic events happened in the DOACs with sulfonylureas and sulfonylureas alone groups, respectively (incidence rates =14.8 per 1,000 person-years and 23.7 per 1,000 person-years, respectively). The risk of developing hypoglycemia was again lower for individuals receiving DOACs with sulfonylureas compared to individuals receiving sulfonylureas alone (HR 0.54; 95% CI, 0.27–1.10, *P* = 0.091). However, this was not statistically significant (Table 3). Kaplan–Meier curves for the incidence of hypoglycemia of Analysis 2 are shown in Figure 3.

### Sensitivity Analysis

The results of the sensitivity analyses are presented in Supplementary Tables S2–S4. When re-analyzing the data taking into account only individuals who received subsequent prescriptions within no more than 90-days, the risk of hypoglycemia was higher in individuals receiving warfarin with sulfonylureas compared to individuals receiving sulfonylurea alone (HR 1.14; 95% CI, 1.01–1.30, *P* = 0.032), Supplementary Table S2. However, the results of the matching analysis based on 90-days grace period showed non-statistically significant results between both groups (HR 1.10; 95% CI, 0.71–1.22, *P* = 0.634), Supplementary Table S2.

Results from the inverse probability of treatment weighting (IPTW) were similar to main analyses. Individuals had a higher risk of hypoglycemia by 24% when receiving warfarin with sulfonylureas compared to individuals receiving sulfonylureas alone (HR 1.24; 95% CI, 1.20–1.25, *P* < 0.0001) (Supplementary Table S3). The results of IPTW were different from main analysis when comparing individuals receiving DOACs and sulfonylureas concomitantly against individuals receiving sulfonylureas alone. The risk of hypoglycemia was again lower for individuals receiving DOACs and sulfonylureas, but significant in the IPTW analysis (HR 0.56; 95% CI, 0.36–0.90, *P* = 0.011). Results from the inverse probability of treatment weighting (IPTW), including PS trimming, are presented in Supplementary Table S3.

Results from multiple imputations were also similar to the main analyses, demonstrating warfarin with sulfonylurea treatment was associated with a higher risk of hypoglycemia compared to sulfonylureas alone (HR = 1.08; 95% CI, 1.03–1.13; *p* = <0.001), Supplementary Table S4.

## Discussion

In this large population-based study in UK primary care, we investigated the association between the concurrent use of OACs and sulfonylureas and the risk of hypoglycemia in individuals with T2DM. This study found that warfarin was associated with 38% increased risk of hypoglycemia when used with sulfonylureas concurrently in individuals with T2DM. We found no evidence of an association between the use of DOACs and sulfonylureas concurrently and the risk of hypoglycemia. The study results support the hypothesis that warfarin is associated with an increased risk of hypoglycemia when given concomitantly with sulfonylureas in individuals with T2DM.

### Comparison With Other Studies

There are limited studies that explored the safety of the concurrent use of warfarin with sulfonylureas. Previous RCTs focused on younger population with fewer number of comorbidities. While the probability of adverse events was more common among elderly. This could be related to the difference in excretion rates between different age groups which is vital to maintain correct therapeutic levels (34).

The findings of increased risk of hypoglycemia among the use of warfarin with sulfonylureas are consistent with two previous large database studies using the Medicare claims and Medicaid programs in the United States (11, 12). Romley et al. reported that the concurrent use of warfarin and sulfonylureas was associated with a 22% higher risk for hypoglycemia (11) in a cohort study of 465,918 individuals. Similarly, in a self-controlled case series design, Nam et al. reported an elevated rate of serious hypoglycemia when warfarin was given concomitantly with sulfonylureas (glipizide, glyburide, glyburide) (12). However, in this study, we included DOAC use and had a longer follow-up than the other two studies.

Our findings indicated that there is no evidence of an association found between the use of DOACs and sulfonylureas concurrently and the risk of hypoglycemia. We suggest that our estimate did not reach the significant level due to the small sample size in the compared group and ultimately number if events. DOAC therapy has multiple advantages over warfarin therapy, which include lack of the need for regular monitoring of the degree of anticoagulation and wider therapeutic range (35, 36).

In this study, there was a difference in the incidence of hypoglycemia between the two groups (sulfonylurea alone group in cohort 1 vs. sulfonylurea alone group in cohort 2). However, a possible explanation for the difference in the incidence of hypoglycemia may be due to high proportion of multiple antidiabetic medication use in the sulfonylurea alone group in cohort 2 (81.3%). Use of multiple antidiabetic medication is a well-known risk factor for hypoglycemia and could be the reason for the difference in the incidence of hypoglycemia.

### Potential Mechanisms

The underlying mechanism of action for the concurrent use of warfarin with sulfonylureas and increased risk of hypoglycemia is unclear. Previous pre-clinical hypotheses suggest some potential mechanisms for this association through a drug-drug interaction between the warfarin and sulfonylureas. First, there may be a displaced protein binding mechanism, where interaction between warfarin and sulfonylureas may occur on the site of the protein binding, and thus warfarin may enhance the plasma concentration of sulfonylureas in the blood, and hence increase its activity and risk of hypoglycemia (10). However, previous studies and reviews have described this mechanism of drug interaction to be overestimated and not to have meaningful clinical effects, as it only applies to data from *in vitro* studies, or to drugs that are given through loading intravenous doses (37, 38). Second, a drug-drug interaction through inhibition of the cytochrome CYP2C9 hepatic metabolic pathway has also been suggested. Warfarin, glimepiride, and glipizide are all largely metabolized by hepatic cytochrome CYP2C9 (10), and therefore, warfarin may limit the rate at which the sulfonylurea can be metabolized in the liver (39). This mechanism may explain the findings of this study, especially the fact that widely used drug references warn that the concurrent use of warfarin with sulfonylureas may increase the risk of bleeding (40). However, no previous human studies exist to validate this hypothesis, and future studies to investigate this association are needed.

Furthermore, pre-clinical studies have suggested that osteocalcin which is one of the important bone proteins produced by the bone (41), is involved in the metabolism of glucose and insulin sensitivity through the process of bone mineralization and formation, which requires high energy (42). It has been postulated that the uncarboxylated form of osteocalcin enhances the glucose tolerance by the beta cells in the pancreatic islets and increases the insulin sensitivity in peripheral tissues (42). However, this function is mainly dependent on Vitamin K, and therefore, administration of warfarin may block the activation of the carboxylation forum of osteocalcin and thus increase the uncarboxylated forum in the plasma (42). *In vitro* studies have suggested stimulation in the production of undercarboxylated osteocalcin by insulin through a positive feedback mechanism between the pancreas, adipose tissue and bone, which in turn enhances insulin production and sensitivity (43).

### Meaning of the Study

Warfarin therapy is indicated as a prophylaxis and treatment for a wide range of life-threatening health conditions including venous thrombosis, pulmonary embolism, and thromboembolic complications associated with AF and cardiac valve replacement. Additionally, warfarin therapy proven to reduce the risk of death, recurrent MI and thromboembolic events (44). Nonetheless, warfarin therapy needs precise dosing regimen and follow-up by the patients and their healthcare professionals due to its associated potential drug-drug interaction and contraindications. Given the fact that diabetes and AF are highly prevalent, and that antidiabetic and anticoagulant medications are largely prescribed concomitantly (14, 15), this urge us to weight the benefit-risk ratio in the case of the concurrent use of antidiabetic and anticoagulant medications therapy and to find the optimal combination therapy for patients (45). Several important drug references have warned that warfarin may have some drug interactions when given with sulfonylureas including the possibility of bleeding, as sulfonylureas may increase the effect of warfarin. However, this possible drug interaction has not been studied extensively or appreciated in the literature and future studies are needed to investigate the association of the concurrent use of warfarin and sulfonylureas and the risk of bleeding. This study provides the first evidence for this drug interaction of two widely used medications in the UK, and it is also consistent with the findings from the previous studies in the US. In addition, this study found an insignificant reduced risk of hypoglycemia when DOACs are used concurrently with sulfonylureas.

Diabetes is associated with cardiac structural and functional alterations which may affect the outcomes of patients with DM and therefore, choosing efficacious treatment regimen is important to reduce the risk of complications (46). Previous studies reported controversial results regarding the use of sulfonylureas in patients with DM and cardiac complications (46). In addition, current major Guidelines for diabetes recommend the use of metformin and sodium-glucose co-transporter 2 (SGLT2) inhibitors for patients with DM and cardiac complications, as they have showed better outcomes and improvements, however, studies to investigate the concurrent use of metformin or SGLT2 with warfarin and/or DOACs are lacking, and we urge for future studies to investigate this association.

### Implication of the Study

Individuals with T2DM receiving OACs are older and more susceptible to polypharmacy and drug-drug interactions (14, 15). Doctors and clinical pharmacists must be vigilant when prescribing warfarin with sulfonylureas and must be alert to both immediate and delayed-onset hypoglycemia when prescribing this drug combination. Clinical surveillance, frequent blood glucose measurements, INR monitoring, diet changes and patient education may be necessary to reduce the risk of hypoglycemia if individuals are prescribed these medications together (47, 48).

Additionally, given that DOACs are widely available nowadays, these medications may be an alternative therapy when OACs and sulfonylureas are indicated in individuals with T2DM. DOACs have a more predictable pharmacokinetic profile and have less drug-drug interactions.

This research has also highlighted a possible protective effect of DOACs against hypoglycemia when prescribed with sulfonylureas; however, the sample size was small, and we did not have a long follow-up time. Therefore, considering these results in the context of the currently available literature, we underline the need for future research with a longer follow-up time, and large sample sizes to examine the association of DOACs and sulfonylureas and the risk of hypoglycemia or bleeding in individuals with T2DM.

### Strengths and Limitations of the Study

Our study has several strengths. Firstly; this study had a longer follow up period compared to the previous studies (11, 12). Secondly, this study used the population representative data from the UK primary care database—THIN. It is therefore, reasonable to assume that our findings are generalized and may broadly reflects real world practice in the UK and the world. Thirdly, we used advanced statistical method (i.e., PS matching and IPTW) to address the measured confounder issue at baseline. In addition, to confirm the robustness of our results, we conducted several sensitivity analyses that suggest our results are robust, including multiple prescriptions grace periods, IPWT and multiple imputations.

This study has some limitations. Firstly, this study is an observational cohort study, and unlike RCTs, the residual confounding cannot be excluded. Secondly, due to the emergency nature of the outcomes of interest (hypoglycemia), we did not have access to hospital data, also, mild hypoglycemia may not be reported to the doctors, and this could lead to underestimations of the cases. Thirdly, in our study we were unable to adjust or match for laboratory parameters. Despite that wide range of confounders were adjusted for in our analyses, which is potentially justified, this could increase the risk of the model fit being affected when there is a small sample size or frequency of outcomes. THIN is an administrative database and therefore, data on medication adherence, the actual ingestion of medications or diet is lacking. However, we tackled this by conducting a sensitivity analysis to account only for prescription refills of fewer than 90 days.

## Conclusion

In summary, the study found that warfarin was associated with an increased risk of hypoglycemia when given concomitantly with sulfonylureas in individuals with T2DM. Concurrent use of DOACs with sulfonylureas however was associated with an insignificant reduced risk of hypoglycemia. The decision to prescribe warfarin with coexistent sulfonylureas to individuals with T2DM should be carefully evaluated in the context of other risk factors of hypoglycemia, and the availability of alternative medications. Future studies are needed to validate the finding of the association of DOACs and sulfonylureas and the risk of hypoglycemia on larger sample size and longer follow-up periods.

## Data Availability Statement

The datasets presented in this article are not readily available because the data can only be accessed through permission from THIN only. Requests to access the datasets should be directed to l.wei@ucl.ac.uk.

## Ethics Statement

This study was reviewed and scientific approval was obtained by IQVIA Scientific Review Committee in 2018 (18THIN046). Written informed consent for participation was not required for this study in accordance with the national legislation and the institutional requirements.

## Author Contributions

Concept and design, and had full access to all the data in the study and take responsibility for the integrity of the data and the accuracy of the data analysis: HA, IW, and LW. Acquisition, analysis, or interpretation of data: HA, IW, AB, PM, CW, and LW. Drafting of the manuscript: HA, IW, AB, AN, CW, and LW. Statistical analysis: HA, PM, and LW. Administrative, technical, or material support: HA. Study supervision: IW, CW, and LW. Critical revision of the manuscript for important intellectual content: All authors. All authors contributed to the article and approved the submitted version.

## Funding

HA project was supported by a scholarship from the Saudi Arabian Ministry of Higher Education.

## Conflict of Interest

The authors declare that the research was conducted in the absence of any commercial or financial relationships that could be construed as a potential conflict of interest.

## Publisher's Note

All claims expressed in this article are solely those of the authors and do not necessarily represent those of their affiliated organizations, or those of the publisher, the editors and the reviewers. Any product that may be evaluated in this article, or claim that may be made by its manufacturer, is not guaranteed or endorsed by the publisher.
