# Corrigendum: Concurrent use of oral anticoagulants and sulfonylureas in individuals with type 2 diabetes and risk of hypoglycemia: a UK population-based cohort study

**DOI:** 10.3389/fmed.2023.1337712

**Published:** 2024-01-05

**Authors:** Hassan Alwafi, Ian C. K. Wong, Abdallah Y. Naser, Amitava Banerjee, Pajaree Mongkhon, Cate Whittlesea, Alaa Alsharif, Li Wei

**Affiliations:** ^1^Research Department of Practice and Policy, School of Pharmacy, University College London, London, United Kingdom; ^2^Faculty of Medicine, Umm Al Qura University, Mecca, Saudi Arabia; ^3^Centre for Safe Medication Practice and Research, Department of Pharmacology and Pharmacy, Li Ka Shing Faculty of Medicine, The University of Hong Kong, Pokfulam, Hong Kong SAR, China; ^4^Department of Pharmacy, The University of Hong Kong - Shenzhen Hospital, Shenzhen, China; ^5^Laboratory of Data Discovery for Health, Hong Kong Science Park, Pak Shek Kok, Hong Kong SAR, China; ^6^Centre for Medicines Optimisation Research and Education, University College London Hospitals National Health Service (NHS) Foundation Trust, London, United Kingdom; ^7^Department of Applied Pharmaceutical Sciences and Clinical Pharmacy, Faculty of Pharmacy, Isra University, Amman, Jordan; ^8^Institute of Health Informatics, University College London, London, United Kingdom; ^9^Department of Cardiology, University College London Hospitals NHS Trust, London, United Kingdom; ^10^Department of Cardiology, Barts Health NHS Trust, London, United Kingdom; ^11^Department of Pharmacy Practice, School of Pharmaceutical Sciences, University of Phayao, Phayao, Thailand; ^12^Pharmacoepidemiology and Statistics Research Center (PESRC), Faculty of Pharmacy, Chiang Mai University, Chiang Mai, Thailand; ^13^Department of Pharmacy Practice, College of Pharmacy, Princess Nourah bint Abdulrahman University, Riyadh, Saudi Arabia

**Keywords:** oral anticoagulants, hypoglycemia, sulfonylureas, diabetes mellitus, United Kingdom, drug-drug interactions

In the published article, there was a mistake in the affiliation for “Alaa Alsharif.” The affiliation for “Alaa Alsharif” was displayed as “Research Department of Practice and Policy, School of Pharmacy, University College London, London, United Kingdom.” The correct affiliation is “Department of Pharmacy Practice, College of Pharmacy, Princess Nourah bint Abdulrahman University, Riyadh, Saudi Arabia.”

The authors apologize for this error and state that this does not change the scientific conclusions of the article in any way. The original article has been updated.

