# Supplementary material for: Concurrent Use of Oral Anticoagulants and Sulfonylureas in Individuals With Type 2 Diabetes and Risk of Hypoglycemia: A UK Population-Based Cohort Study
Source: Front Med (Lausanne). 2022 Aug 23;9:893080. doi: 10.3389/fmed.2022.893080 (PMC9445245; doi:10.3389/fmed.2022.893080)
Supplement: Supplementary file 1 [file Table_1.DOCX]

**SUPPLEMENTAL MATERIAL**

**Article title:** Concurrent use of oral anticoagulants and sulfonylureas in individuals with type 2 diabetes and risk of hypoglycaemia: A UK population-based cohort study.

# Short-running title: Oral anticoagulants and sulfonylureas and risk of hypoglycaemia

# Authors information: Hassan Alwafi^1,2^; M.D, Ian CK Wong^1,3,4^; PhD, Amitava Banerjee^5,6,7^, PhD, Pajaree Mongkhon^8,9^; PhD, Abdallah Y Naser^10^; PhD, Cate Whittlesea^1^; PhD, Alaa Alsharif^1^; MSc, Li Wei^1^; PhD

# Authors affiliation:

^1^Research Department of Practice and Policy, School of Pharmacy, University College London, London, United Kingdom

^2^Faculty of Medicine, Umm Al Qura University, Mecca, Saudi Arabia.

^3^Centre for Safe Medication Practice and Research, Department of Pharmacology and Pharmacy, Li Ka Shing Faculty of Medicine, The University of Hong Kong, Hong Kong.

^4^The University of Hong Kong - Shenzhen Hospital, 1, Haiyuan 1st Road, Futian District, Shenzhen, Guangdong, China.

^5^Institute of Health Informatics, University College London, London, UK

^6^Department of Cardiology, University College London Hospitals NHS Trust, London, UK.

^7^Department of Cardiology, Barts Health NHS Trust, London, UK

^8^Department of Pharmacy Practice, School of Pharmaceutical Sciences, University of Phayao, Thailand.

^9^Pharmacoepidemiology and Statistics Research Center (PESRC), Faculty of Pharmacy, Chiang Mai University, Chiang Mai, Thailand.

^10^Department of Applied Pharmaceutical Sciences and Clinical Pharmacy, Faculty of Pharmacy, Isra University, Amman, Jordan.

# Corresponding author

# Professor Li Wei

# UCL School of Pharmacy

# 29-39 Brunswick Square

# London, WC1N 1AX

# T: 020 7874 1275

# E: [l.wei@ucl.ac.uk](mailto:l.wei@ucl.ac.uk)

**Tables (3)**

**Table S1.** Patient’s characteristics among the cohort of second analysis (sulfonylureas and DOACs versus sulfonylureas only).

**Table S2.** Cox proportional hazard (Un-adjusted/Adjusted/Matched) for risk of hypoglycaemia 90-days.

**Table S3**. Cox proportional hazard results from IPWT analysis.

**Table S4**. Cox proportional hazard results from multiple imputation method (m=25)

**Table S5.** Number of events, incidence rates and crude HR, for risk of hypoglycaemia among different types of sulfonylureas.

**Table S6**. Number of events, incidence rates and HR, for risk of hypoglycaemia for the matched cohort among different types of sulfonylureas.

Table S1. Patient’s characteristics among the cohort of second analysis (sulfonylureas and DOACs versus sulfonylureas only)

| Variable | Before propensity scree matching  No. (%) of participant | | | | After propensity scree matching  No. (%) of participant | | |
| --- | --- | --- | --- | --- | --- | --- | --- |
|  | All  (n=77,296) | Sulfonylureas + DOACs  (n= 1,045) | Sulfonylureas  (n= 76,251) | Crude ASD | Sulfonylureas + DOACs  (n= 1,027) | Sulfonylureas  (n= 1,027) | Matched ASD |
| Demographics | | | | | | | |
| Age mean (SD) | 63.9 (SD13.1) | 73 (10.2) | 63.7 (13.0) | 0.793 | 73.3 (10.3) | 74.3 (11.1) | 0.106 |
| Male, n (%) | 45,112 (58.3) | 645 (61.7) | 44,467 (58.3) | 0.070 | 632 (61.5) | 608 (59.2) | -0.048 |
| BMI |  |  |  | 0.088 |  |  | 0.080 |
| BMI < 25 | 10910 (14.1) | 124 (11.8) | 10786 (14.1) | - | 120 (11.7) | 133 (13.0) |  |
| BMI 25-30 | 25143 (32.5) | 322 (30.8) | 24821 (32.5) | - | 318 (31.0) | 346 (33.7) |  |
| BMI ≥ 30 | 41243 (53.3) | 599 (57.3) | 40644 (53.3) | - | 589 (57.3) | 548 (53.4) |  |
| Smoking |  |  |  | 0.111 |  |  | 0.015 |
| Non-smokers | 64954 (84.0) | 918 (88.0) | 64036 (84.0) | - | 903 (88.0) | 908 (88.4) | - |
| Smokers | 12342 (16.0) | 127 (12.0) | 12215 (16.0) | - | 124 (12.0) | 119 (11.6) | - |
| Alcohol |  |  |  | 0.079 |  |  | 0.044 |
| Non-drinker | 24607 (32.0) | 371 (35.5) | 242316 (32.0) | - | 364 (35.4) | 386 (37.6) | - |
| Drinker | 52689 (68.0) | 674 (64.5) | 52015 (68.0) | - | 663 (64.5) | 641 (62.4) | - |
| Townsend |  |  |  | 0.087 |  |  | 0.055 |
| 1 (Least deprived) | 14950 (19.3) | 195 (18.7) | 14755 (19.3) | - | 190 (18.5) | 184 (18.0) | - |
| 2 | 15403 (20.0) | 199 (19.0) | 15204 (19.9) | - | 196 (19.0) | 201 (19.6) | - |
| 3 | 17042 (22.0) | 238 (22.8) | 16804 (22.0) | - | 233 (22.7) | 214 (20.9) | - |
| 4 | 16654 (21.5) | 204 (19.5) | 16450 (21.6) | - | 201 (19.6) | 216 (21.0) | - |
| 5 (Most deprived) | 13247 (17.1) | 209 (20.0) | 13038 (17.1) | - | 207 (20.1) | 212 (20.6) | - |
| Comorbid conditions, n (%) | | | | | | | |
| CVDs | 4308 (5.5) | 185 (17.7) | 4123 (5.4) | 0.393 | 175 (17.0) | 194 (18.9) | -0.048 |
| Hypertension | 46371 (60.0) | 776 (74.2) | 45595 (60.0) | 0.311 | 763 (74.3) | 778 (75.7) | -0.034 |
| Stroke/TIA | 5700 (7.3) | 229 (22.0) | 5471 (7.1) | 0.428 | 219 (21.3) | 235 (22.9) | 0.038 |
| Bleeding | 13882 (18.0) | 285 (27.3) | 13597 (18.0) | 0.227 | 277 (27.0) | 310 (30.2) | -0.071 |
| Hyperlipidaemia | 17699 (23.0) | 230 (22.0) | 17469 (22.9) | 0.022 | 229 (22.3) | 245 (22.8) | -0.037 |
| AF | 2313 (3.0) | 645 (61.7) | 1668 (2.1) | 1.658 | 627 (61.0) | 621 (60.5) | 0.012 |
| DVT | 2215 (2.9) | 162 (15.5) | 2053 (2.7) | 0.457 | 159 (15.5) | 201 (19.6) | -0.108 |
| Chronic kidney disease | 17295 (22.4) | 364 (34.4) | 16931 (22.2) | 0.282 | 360 (35.0) | 402 (39.0) | -0.085 |
| COPD | 4176 (5.4) | 120 (11.4) | 4056 (5.3) | 0.224 | 118 (11.5) | 134 (13.0) | -0.048 |
| Hyperglycaemia | 2778 (3.6) | 37 (3.5) | 2741 (3.6) | 0.003 | 36 (33.5) | 33 (33.2) | 0.016 |
| Liver diseases | 527 (0.7) | 10 (1.0) | 517 (0.7) | 0.031 | 10 (1.0) | 11 (0.9) | -0.010 |
| Depression | 21066 (27.3) | 303 (29.0) | 20763 (27.3) | 0.039 | 295 (28.7) | 304 (29.6) | -0.019 |
| Anxiety | 14160 (18.3) | 202 (19.3) | 13958 (18.3) | 0.026 | 199 (19.3) | 201 (19.6) | -0.005 |
| Baseline medication use, n (%) | | | | | | | |
| Aspirin use | 32006 (41.4) | 480 (46.0) | 31526 (41.3) | 0.093 | 476 (46.3) | 526 (51.2) | -0.098 |
| Antiplatelet drugs use | 3644 (4.7) | 172 (16.5) | 3472 (4.5) | 0.396 | 166 (16.1) | 180 (17.5) | -0.036 |
| Beta blockers use | 18316 (23.7) | 659 (63.0) | 17657 (23.1) | 0.880 | 642 (62.5) | 622 (60.5) | 0.040 |
| ACEIs /ARBs use | 47369 (61.2) | 761 (73.0) | 46608 (61.1) | 0.251 | 745 (72.5) | 748 (72.8) | -0.007 |
| Corticosteroids use | 5051 (6.5) | 161 (15.4) | 4890 (6.4) | 0.291 | 154 (15.0) | 156 (15.2) | -0.005 |
| Multiple antidiabetic medications use (intensification) | 65600 (85.5) | 875 (83.7) | 64725 (85.4) | 0.032 | 857 (83.4) | 835 (81.3) | 0.056 |

Table S2. Cox proportional hazard (Un-adjusted/Adjusted/Matched) for risk of hypoglycaemia 90-days

| **Exposure group** | **Un-adjusted hazard-ratio (95% CI)** | **Adjusted hazard-ratio (95% CI)** | **Matched by propensity score (95%CI)** |
| --- | --- | --- | --- |
| Warfarin+ sulfonylureas | 1.14  (1.01- 1.30) | 1.00  (0.85- 1.15) | 1.10  (0.71- 1.22) |
| Ref=Sulfonylurea only |  |  |  |
| DOACs+ sulfonylureas | 1.05  (0.70- 1.60) | 0.79  ( 0.52- 1.21) | 0.80  (0.44- 1.40) |
| Ref=Sulfonylurea only |  |  |  |

Table S3. Cox proportional hazard results from IPWT analysis

| **Exposure group** | **IPWT (95% CI)** | **IPWT (95%CI)**  **1%-99% percentile** |
| --- | --- | --- |
| Warfarin+  sulfonylureas | 1.24  (1.20-1.25) | 1.12  (1.01- 1.24) |
| Ref=Sulfonylurea only |  |  |
| DOACs+  sulfonylureas | 0.56  (0.36- 0.90) | 0.39  (0.244- 0.60) |
| Ref=Sulfonylurea only |  |  |

Table S4. Cox proportional hazard results from multiple imputation method (m=25)

| **Exposure group** | **Crude HR (95% CI)** | **P-value** | **Matched HR (95% CI)** | **P-value** |
| --- | --- | --- | --- | --- |
| Warfarin+  Sulfonylureas | 1.22 (1.13-1.32) | <0.0001 | 1.08 (1.03-1.13) | <0.0001 |
| Ref=Sulfonylurea only |  |  |  |  |
| DOACs+  Sulfonylureas | 0.9 (0.59-1.36) | 0.593 | 0.54 (0.46 -0.62) | <0.0001 |
| Ref=Sulfonylurea only |  |  |  |  |

**Table S5. Number of events, incidence rates and crude HR, for risk of hypoglycaemia among different types of sulfonylureas.**

| Exposure group | No. of event | Person-years at risk, year | IR, per 1000  person-years  (95% CI) | Crude HR  (95% CI) |
| --- | --- | --- | --- | --- |
| gliclazide+warfarin  (n= 8266) | 525 | 29883.39 | 17.6 | 1.23  (1.12- 1.34) |
| gliclazide only  (n= (n=76527) | 6570 | 476681.92 | 13.8 | 1.00 |
| glimepiride+warfarin  *(n= 924) | 46 | 3472.34 | 13.2 | 0.66  (0.39- 1.11) |
| glimepiride only *  (n= (11306) | 859 | 65886.83 | 13.0 | 1.00 |
| glipizide+warfarin  (n=463) | 19 | 1676.22 | 11.0 | 0.98  (0.61-1.55) |
| glipizide only  (n=5266) | 372 | 34547.46 | 13.3 | 1.00 |
| glyburide +warfarin  (n= 423) | 38 | 1699.72 | 22.3 | 1.82  (1.30 2.52) |
| glyburide only  (n= 5865) | 608 | 46533.57 | 13.0 | 1.00 |

**Table S6. Number of events, incidence rates and HR, for risk of hypoglycaemia for the matched cohort among different types of sulfonylureas.**

| Exposure group | No. of event | Person-years at risk, year | IR, per 1000  person-years  (95% CI) | Matched HR  (95% CI), p-value |
| --- | --- | --- | --- | --- |
| gliclazide+warfarin (n=4517) | 250 | 13909.10 | 18.7 | 1.13  (0.89- 1.45) |
| gliclazide only (n=4517) | 284 | 17794.84 | 15.7 | 1.00 |
| glimepiride +warfarin  *(n=435) | 18 | 1674.00 | 11.0 | 1.00  (0.74- 1.35) |
| glimepiride only * (n=435) | 27 | 2034.98 | 13.2 | 1.00 |
| glipizide +warfarin  (n=215) | 8 | 802.41 | 10.0 | 0.83  (0.25 -2.73) |
| glipizide only  (n=215) | 14 | 1252.84 | 11.1 | 1.00 |
| glyburide +warfarin  (n= 247) | 25 | \|  \|  \| \| --- \| --- \|   1042.41 |  | 2.17  (0.82- 5.70) |
| glyburide only  (n= 247) | 17 | 1577.31 |  | 1.00 |
